# The feasibility of Gazefinder under 12 months of age infants

**DOI:** 10.1038/s41598-021-89585-7

**Published:** 2021-05-11

**Authors:** Shuntaro Fukushima, Tomoo Takahashi, Kazuki Tsukamoto, Misaki Matsumura, Ryo Takigawa, Yasuo Sakai, Sokichi Maniwa, Lynne Murphy, Takeshi Taketani

**Affiliations:** 1 Division of Pediatrics, Ohchi Municipal Hospital, 3848-2, Nakano Ohnan-cho, Ohchi, Shimane 696-0102 Japan; 2 Department of Pediatrics, Shimane University Faculty of Medicine, 89-1, Enya-cho, Izumo, Shimane 693-8501 Japan; 3 Department of Rehabilitation Medicine, Shimane University Faculty of Medicine, Izumo, Shimane Japan; 4 Department of Medical English Education, Shimane University Faculty of Medicine, Izumo, Shimane Japan

**Keywords:** Developmental biology, Neuroscience, Medical research, Neurology, Signs and symptoms

## Abstract

Eye-tracking to evaluate gaze patterns has developed as an assessment tool for children with autism spectrum disorder (ASD). Gazefinder is one of Eye-tracking devices and few studies have investigated whether it can measure the gaze data of infants under 12 months of age. We conducted a prospective cross-sectional study from April 2019 to March 2020 in a periodic health checkup in Ohchi County, Shimane, Japan. Participants included infants between 4 and 11 months of age who were not suspected the presence of developmental problems. Ninety-three participants’ datapoints were analyzed. The mean age was 6.5 months and mean developmental quotient was 88%. The mean fixation time percentage of all sequences was 81.0% (standard deviation; 4.4), and there was no significant difference in each age group. Infants in all groups showed a significantly higher predilection for eyes than for mouths. There was a positive association of age with human gaze and a negative association with geometric gaze. Moreover, we confirmed that joint attention skills were enhanced in accordance with their growth process. The eye-tracking data were almost corresponding to previous studies’ data of infant with typical development and Gazefinder could be applied to infants starting at 4 months of age.

## Introduction

Autism spectrum disorder (ASD) is a neurodevelopmental condition characterized by “deficits in social communication and social interaction” and “restricted, repetitive patterns of behavior, interests, or activities”^1^. In Japan, the prevalence of individuals with ASD is approximately 1% and an upward trend has been reported^2^. Early identification and treatment of children with ASD are regarded as important factors for improving their lifetime outcomes^3^. The efficacy of ASD-specific interventions for children as early as 18 months of age has been reported^4,5^.

Early symptoms, which can be seen in infants with ASD in the first year of life, include reduced motor control, attention, gazing of faces, and emotional regulation, before the development of overt social communication impairments and repetitive behaviors^6^. Eye-tracking to evaluate gaze patterns has advantages as an assessment tool for children with ASD. It can be applied to young children, possibly leading to earlier identification of gaze risk, and provides quantitative data that can be used potentially as biomarkers that indicate abnormal visual attention and possible information processing deficits^7–18^. Several eye-tracking devices have been developed, and gaze patterns of ASD and Typical Development (TD) have also been documented in various age groups^7–22^. Gazefinder (JVCKENWOOD Corporation, Japan), one of the eye-tracking devices, does not require goggles or head restraints, and needs a short measurement time of 2 min. Several studies have shown its efficacy from children to adults^13–15,19,20^. Although there have been several reports of gaze patterns in infants under 12 months by other eye-tracking devices^7–12,21,22^, there were few studies adopting them for ASD screenings, because of the lack of uniformity in protocols and diagnostic methods. Moreover, the problems with eye tracking in infants include underdeveloped visual acuity, easy distraction, and difficulty in obtaining cooperation for the test, which may prevent the acquisition of valid data. If it can be confirmed that Gazefinder overcome these problems, it will be possible to collect a large amount of gaze patterns data, which may contribute to the establishment of protocols and diagnostic criteria for the early detection of children with ASD. periodic health checkups are widely and equally available to infants and appropriate as an opportunity for future eye-tracking screening. Most of the infants who undergo periodic health checkups are TD, and their gaze patterns data are important for comparison with ASD. Prior to the study in infants with ASD, this study was conducted as a pilot study to investigate the feasibility of using Gazefinder in infants with TD, who were not suspected of having ASD at the time, under 12 months of age.

## Methods

### Study design

We conducted a prospective cross-sectional study from April 2019 to March 2020. Infants between 4 and 11 months of age who underwent a periodic health checkup in Ohchi County, Shimane, Japan were recruited. In this county, public periodic health checkups are conducted twice for infants younger than 1 year (at 4–6 and 7–11 months of age). For infants who received two-times checkups during the period, the gaze patterns were measured in each health checkup.

Participants with the following conditions were excluded: no head control; acute infections; strabismus or congenital visual impairment; brain damage (fetal distress, epilepsy, abuse, head injury, or history of maternal medication during the fetal period) and those who could not continue the examination due to crying or fussiness. Psycho-developmental evaluations were not performed in candidates for this study. However, the candidates were examined by a pediatrician before testing, and if the presence of a developmental disability was suspected, they were excluded from recruitment.

### Eye-tracking device

**Figure 1:**
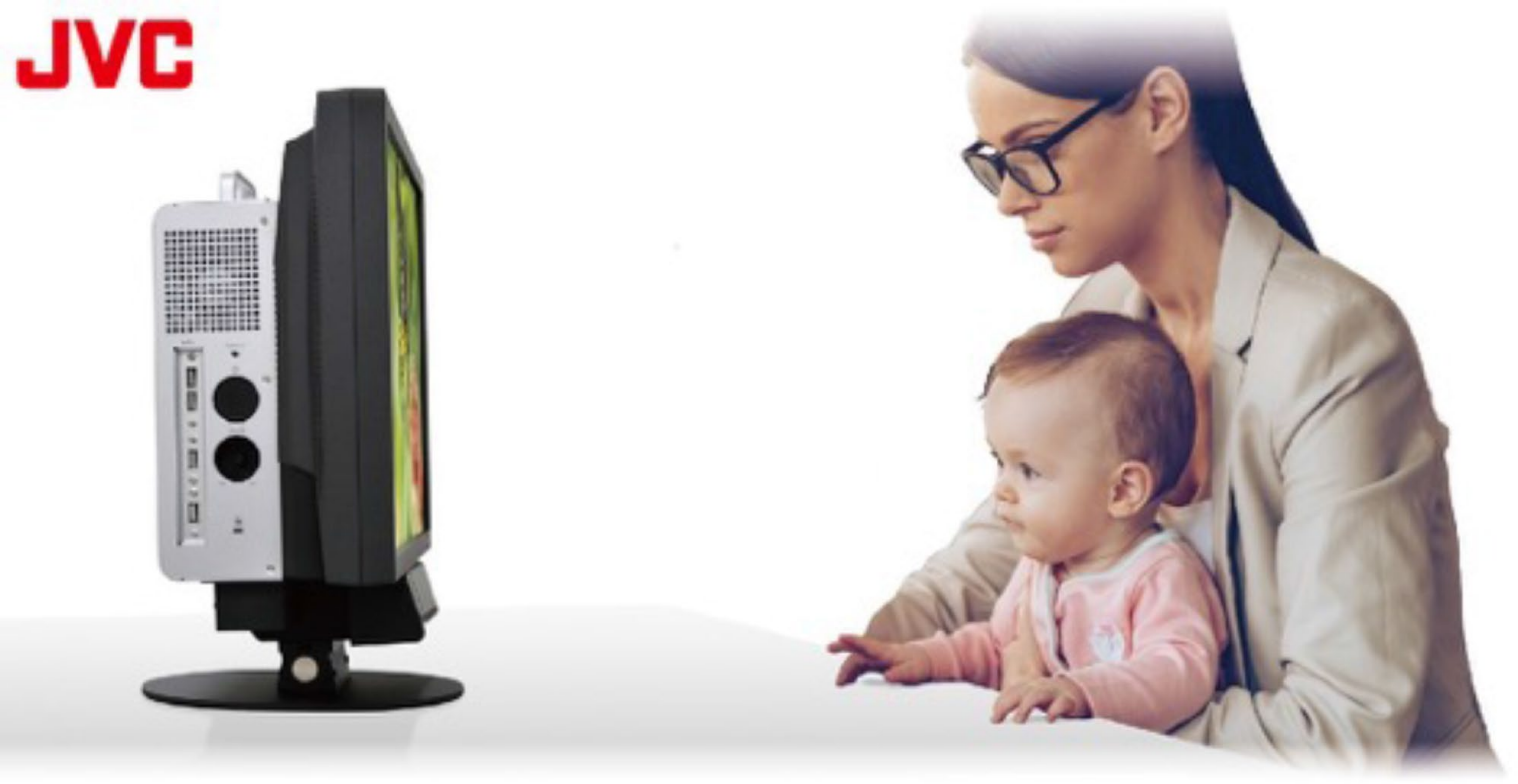
The image of the measurement by Gazefinder.

Gazefinder is an all-in-one eye-tracking system that evaluates the percentage of fixation times allocated to specific objects on a video monitor. Gazefinder used infrared light sources and cameras that were integrated into a 19-inch-thin film transistor monitor (1280 × 1024 pixels). Using corneal reflection techniques, Gazefinder records the X and Y coordinates of each child's eye position at a frequency of 50 Hz. Gaze patterns can be examined by simply looking at the monitor for two minutes after a 15-s calibration of the eye position with a five-point method. The image of the measurement is presented in Fig. 1.

### Stimuli

Nine patterns of short movies were presented, including five movies of a human face (I–V), one movie of biological motion (VI), two movies of human and geometric patterns (VII–VIII); and one movie of joint attention (IX). The human face movie clips consisted of the following: (I) eye blinking (an actress repeatedly opens and closes her eyes for 5 s); (II) mouth moving (an actress repeatedly opens and closes her mouth for 5 s); (III) still image (4 s); (IV) silence (an actress with a still face for 5 s and it appears after the mouth moving movie) and (V) talking (7 s). In movie V, the actress says, “Hello, what is your name?”, and “Let’s play together” in Japanese. Movie VI simultaneously presents upright and inverted biological motions for 11 s. This movie was accompanied by a soundtrack playing the song “Under the Big Chestnut Tree”, with an upright human (moving with the music) and inverted human (moving un-match with the music). Movies VII–VIII consisted of human and geometry presented at the same time and of the same size (20 s) or geometric patterns depicted through a small window in a movie of human (16 s), respectively. The joint attention movie (IX) showed an actress who faces the front and some objects include geometric patterns, and an actress who turns to face one of the objects and points toward it one second later (8 s). Stimulus movies were displayed in a definitive order^15^.

Each movie (I–VIII) contained two target areas (Group A, Group B). Movie IX has three target areas (Group A, Group B and Group C). The target areas were set in movies I-V as Group A (eyes)/Group B (mouth); movie VI as Group A (upright)/Group B (upside down); in movies VII–VIII as Group A (person)/Group B (geometric pattern); and movie IX as Group A (person)/Group B (pointed object)/Group C (geometric pattern).

**Figure 2:**
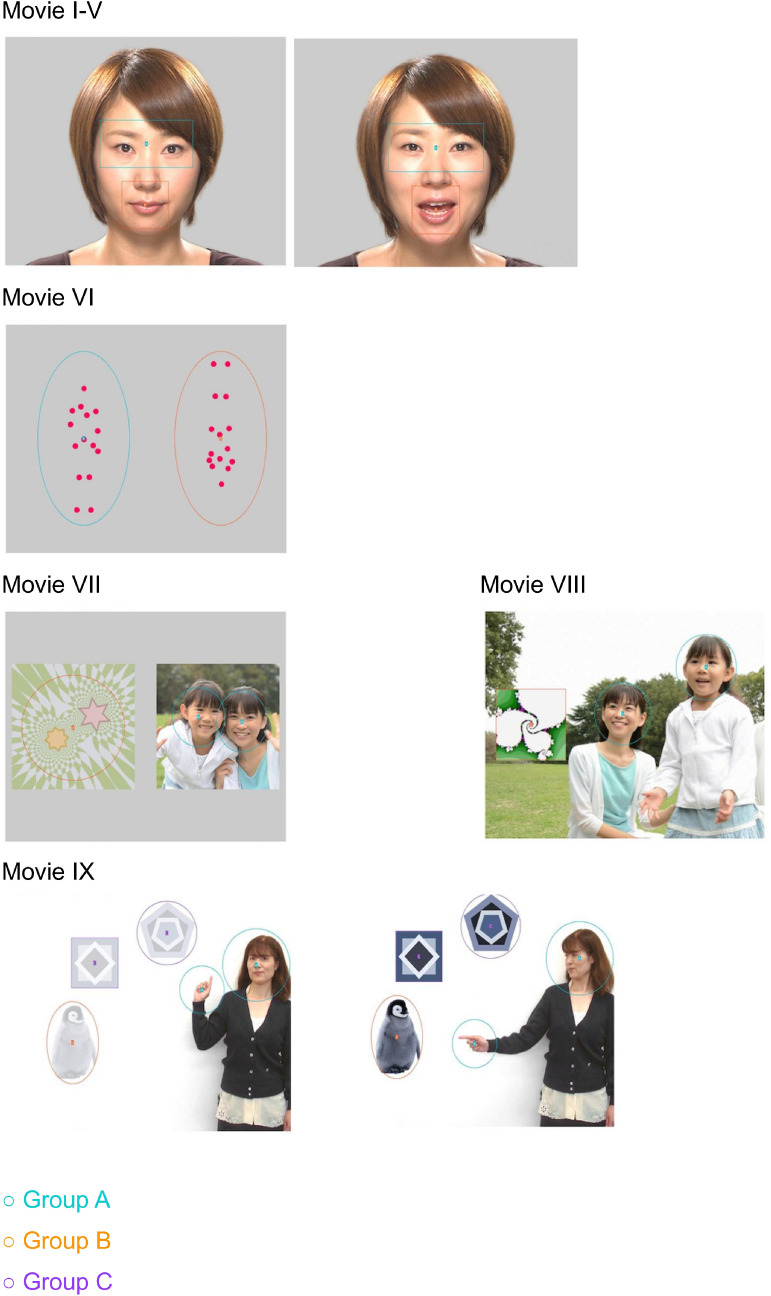
Gazefinder movie samples and their target areas. The human face (Movie I–V) with or without mouth motion; Group A and Group B include the eyes and mouth regions, respectively; Biological motion (Movie VI) Group A and Group B are the upright and inverted images, respectively; Human and geometric patterns (Movie VII and VIII); Group A and Group B are human and geometry, respectively; Joint attention (Movie IX); Group A, Group B and Group C are human, pointed object and geometry areas, respectively.

The movies and target areas were set as default. The fixation percentages allocated to particular areas (Group A, Group B, Group C) were collected automatically. Stimuli movie samples and their target areas are presented in Fig. 2.

### Measurement method

Infants were held forward on their caregiver’s lap and placed approximately 40–50 cm away from the monitor. To prevent distraction, examinations were conducted in a private room, with blinds on the windows, and with the lights in the room on. There were only three persons (the participant, their parent, and an operator) allowed in the room and the operator sat behind the participant and their caregivers.

### Measurement data

We collected data on participants’ sex, age, gestational age, birth weight, and developmental index. The fixation percentages of all sequences (a participant’s total gaze time divided by the total movie sequence time [2 min]), and those allocated to particular areas (Group A, Group B, Group C, and Other areas) were collected. In this study, the fixation percentage of all sequences was regarded as a scale of how long infants can gaze at a monitor, and the fixation percentage allocated to particular areas (Group A–C) was regarded as a scale of the extent that infants can identify the contents of movies, not as cues of ASD screening.

### Criteria for feasibility

In previous research on children between the ages of 1 year and 6 months to 2 years and 2 months^14^, the Modified Checklist of Autism for Toddlers (M-CHAT) and Gazefinder gaze fixation data showed a significant correlation with the Algorithm Diagnosis. In this algorithm, the Youden Index (sensitivity + specificity − 1) was highest when using gaze data that was ≥ 50% of the fixation time percentage of all sequences. Although the age of the subjects differed from our study, we set the criteria of the mean fixation time percentage of all sequences as "at least 50% or more”.

Based on previous reports^3,7–23^, we hypothesized that the gaze patterns for each stimulus movie of Gazefinder would tend to be as follows; preference for eyes in human face (Movie I–V), upright biological motion (Movie VI), humans over geometric patterns (Movie VII) and acquiring joint gaze with aging (Movie IX) in under 12 months infant without ASD. We examined the reproducibility between this hypothesis and the results obtained by Gazefinder.

### Statistical analysis

Using the Enjoji Scale of Infant Analytical Development Test^24^, we evaluated developmental age (months) and calculated participants’ developmental quotient (DQ), which was defined as the developmental age (months) divided by calendar age (months). Initially, we planned to analyze the participants data for each months of age. However, the number of samples was extremely small for some age, and it was considered difficult to conduct appropriate analysis. Then we divided the participants into four age groups (4–5, 6–7, 8–9, and 10–11 months) and investigated differences in the fixation percentages of all sequences between the four groups. The data of the four groups were not normally distributed and had a small sample size; therefore, we used a non-parametric test (Kruskal–Wallis test). Mann–Whitney test was used to compare the data by gender.

We also examined differences in the fixation percentages allocated to particular areas (Group A, Group B, and Group C) in each movie. The data were not normally distributed in all movies, and we used a nonparametric test for comparison: Wilcoxon rank sum test for movies I–VII and Friedman's test for movie IX. Next, we examined the transition of the fixation percentages allocated to the particular areas by the four age groups. The data were not normally distributed and a Kruskal–Wallis test was used for analysis. Subsequently, the data that showed statistical significance were subjected to multiple comparisons (pairwise test). Moreover, the data in subjects who underwent two periodic health checkups during the study period were analyzed. Wilcoxon rank-sum test was performed, because the number of samples was small and the distribution was not normal. Statistical analyses were conducted using EZR^25^. This was a pilot study using Gazefinder on infants under 12 months of age, and all mean values were presented as representative values for all data, for additional studies in the future.

### Ethics approval

The study was conducted according to relevant guidelines and regulations (Declaration of Helsinki) and was approved by the Shimane University Institutional Committee on Ethics, study number: 3367. Registered 10 October 2018, https://rinken.shimane-u-tiken.jp/files/original/20190430135328390cbfcb41b.pdf.

### Consent to participate

We provided information about the study to the participants’ caregivers in advance and obtained informed consent for participate and publication of their data in writing at the time of examination.

## Results

### Participants

A total of 106 infants between 4 and 11 months of age were enrolled in the study. Thirteen participants were excluded (sleeping [n = 3], fussy [n = 8], strabismus [n = 1], and without head control [n = 1]). Twenty infants underwent two-times health checkups over time during the study period, and their gaze patterns were measured once each time. In total, 93 individual datasets were included in the study (45 [48%] males and 48 [52%] females). The mean age of patients was 6.5 (SD, 2.1) months. The mean DQ was 88% (SD, 13.3 range, 58–124%). Clinical data are presented in Table 1.

**Table 1:** Participants characterization data.

	n	Mean (SD)
Data numbers	93	
Months of age		6.5 (2.1)
Gestational age (week)		38.5 (1.9)
Birth weight (g)		3035.9 (478.4)
Sex (male numbers)	45	
Developmental quotient (%) (the Enjoji Scale)		88 (13.3)

*n* number of participants, *SD* standard deviation.

### Gaze fixations

The mean fixation percentage of all sequences was 81.0% (SD, 4.4). The Kruskal–Wallis test showed no significant difference in the fixation time percentage of all sequences between the four age groups (Table 2). There was no significant difference between male 81.0% (SD, 0.14) and female 81.0% (SD, 0.15) (P > 0.5).

**Table 2:** Fixation time percentage of all sequence between four groups.

Months of age	n (%)	Fixation percentage mean (SD)	P-value (Kruskal–Wallis test)
4–5	40 (43.0)	0.83 (0.13)	
6–7	21 (22.6)	0.81 (0.12)	
8–9	23 (24.7)	0.77 (0.16)	
10–11	9 (9.7)	0.80 (0.19)	
All	93	0.81 (0.14)	0.36

*n* number of participants, *SD* standard deviation.

**Figure 3:**
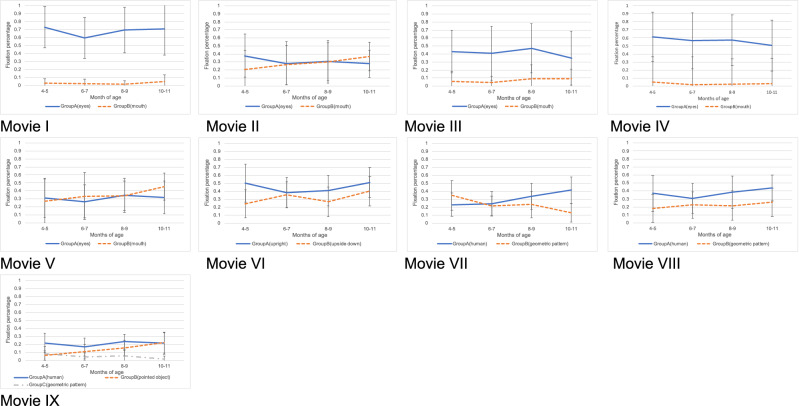
Transition of the fixation percentages allocated to the particular areas in each age group. Line graphs of the percentage fixation times and standard deviations of each age group. Movie I–V; human face; (I) eye blinking; (II) mouth moving; (III) still image; (IV) silent; (V) talking; Movie VI; biological motion; Movie VII; human and geometric patterns (same size) VIII; human and geometric patterns (small window), Movie IX; joint attention. *SD* standard deviation.

Next, the fixation percentages allocated to the particular areas in each movie were compared. Group A had significantly higher fixation percentages than Group B in movies I, III, IV, VI, VIII, and IX (Table 3). In movie IX, the pairwise test showed that the fixation percentages of Group A were highest, followed by Group B, then Group C. We then examined the transitions of the fixation percentage allocated to particular areas by each age group. Significant differences were found in movie VI for Group B (no significant difference in the multiple comparison); movie VII for Group A (4–5 months vs. 10–11 months, p = 0.045); movie VII Group B (4–5 months vs. 10–11 months, p = 0.009); movie IX Group B (4–5 months vs. 8–9 months, p < 0.001; and 4–5 months vs 10–11 months, p = 0.01); and movie IX Group C (no significant difference in the multiple comparison). These data are presented in Table 4 and Fig. 3.

**Table 3:** Fixation percentages allocated to the particular areas in each movie.

Movie	Fixation percentages			P-value	
	Group-A (SD)	Group-B (SD)	Group-C (SD)	Wilcoxon rank sum test	Friedman rank sum test
I	0.68 (0.3)	0.031 (0.063)		< 0.001	
II	0.31 (0.24)	0.28 (0.26)		0.12	
III	0.42 (0.3)	0.072 (0.13)		< 0.001	
IV	0.56 (0.32)	0.029 (0.091)		< 0.001	
V	0.31 (0.23)	0.35 (0.26)		0.77	
VI	0.45 (0.23)	0.32 (0.18)		< 0.001	
VII	0.31 (0.17)	0.23 (0.18)		0.67	
VIII	0.38 (0.21)	0.22 (0.16)		< 0.001	
IX	0.21 (0.12)	0.11 (0.10)	0.064 (0.072)		< 0.001

Movie I–V; human face; (I) eye blinking; (II) mouth moving; (III) still image; (IV) silent; (V) talking; Movie VI; biological motion; Movie VII; human and geometric patterns (same size) VIII; human and geometric patterns (small window), Movie IX; joint attention. *SD* standard deviation.

**Table 4:** Fixation percentage of each age group in I–IX Movies.

Movie	Group	Fixation percentages of each age group (SD)				P-value Kruskal–Wallis test
		4–5	6–7	8–9	10–11	
I	A	0.73 (0.26)	0.6 (0.26)	0.69 (0.29)	0.71 (0.33)	0.61
	B	0.03 (0.06)	0.02 (0.06)	0.02 (0.04)	0.05 (0.08)	0.11
II	A	0.37 (0.27)	0.28 (0.27)	0.3 (0.24)	0.28 (0.17)	0.67
	B	0.2 (0.24)	0.26 (0.24)	0.3 (0.27)	0.37 (0.18)	0.20
III	A	0.43 (0.27)	0.41 (0.34)	0.47 (0.31)	0.35 (0.33)	0.72
	B	0.06 (0.13)	0.05 (0.08)	0.1 (0.17)	0.09 (0.12)	0.31
IV	A	0.61 (0.31)	0.57 (0.35)	0.57 (0.32)	0.5 (0.32)	0.82
	B	0.05 (0.12)	0.02 (0.05)	0.02 (0.06)	0.03 (0.05)	0.28
V	A	0.31 (0.24)	0.27 (0.21)	0.34 (0.21)	0.32 (0.2)	0.76
	B	0.28 (0.28)	0.33 (0.3)	0.34 (0.18)	0.46 (0.17)	0.15
VI	A	0.5 (0.24)	0.38 (0.19)	0.41 (0.19)	0.51 (0.19)	0.10
	B	0.24 (0.18)	0.36 (0.16)	0.27 (0.18)	0.4 (0.18)	0.05
VII	A	0.23 (0.15)	0.25 (0.15)	0.33 (0.16)	0.41 (0.17)	0.05
	B	0.35 (0.18)	0.21 (0.13)	0.23 (0.17)	0.13 (0.12)	0.01
VIII	A	0.37 (0.22)	0.31 (0.19)	0.38 (0.2)	0.44 (0.16)	0.45
	B	0.18 (0.18)	0.23 (0.16)	0.22 (0.18)	0.26 (0.18)	0.46
IX	A	0.22 (0.12)	0.17 (0.11)	0.23 (0.09)	0.09 (0.13)	0.22
	B	0.06 (0.06)	0.11 (0.09)	0.16 (0.1)	0.22 (0.13)	0.00
	C	0.09 (0.09)	0.05 (0.05)	0.06 (0.07)	0.02 (0.03)	0.04

Movie I–V; human face; (I) eye blinking; (II) mouth moving; (III) still image; (IV) silent; (V) talking; Movie VI; biological motion; Movie VII; human and geometric patterns (same size) VIII; human and geometric patterns (small window), Movie IX; joint attention. Group A (eyes)/Group B (mouth) in Movie I–V; Group A (upright)/Group B (upside down) in movies VI; Group A (person)/Group B (geometric pattern) in Movie VII–VIII; Group A (person)/Group B (pointed object)/Group C (geometric pattern) in Movie IX. *SD* standard deviation.

The data of participants who underwent two-times health checkups over time during the study period.

The mean months of age was 4.45 (SD, 0.5) for the first time and 7.55 (SD, 0.74) for the second time. The fixation time percentage of all sequences was 0.81 (SD, 0.16) for the first time and 0.82 (SD, 0.12) for the second time, with no significant difference (p = 0.82). The trend of the gaze patterns of the target area in each stimulus movie was similar to the analysis of all data, and in Movie IX for Group B, the gaze rate tended to increase with age (0.07 in the first time, 0.17 in the second time, p < 0.01).

## Discussion

We investigated the feasibility of using Gazefinder on infants from 4 to 11 months of age. The purpose of this study was to confirm the reciprocity with previously reported gaze patterns of infants under 12 months in measurements with this device. Despite the many insights into ASD development stemming from eye tracking research, difficulty when comparing results from different eye tracking studies of children has been noted^26^. One systematic review^27^, investigated stimuli dimensions and experimental paradigms in eye-tracking research for young children at risk for ASD, highlights variability in eye-tracking protocols and heterogeneity of stimuli as factors that weaken the value of eye-tracking as an objective, reliable screening tool. Although the gaze patterns data itself in our study may not be new to previous studies, it is necessary to confirm that the data obtained by this device and protocol, were consistent with previous reports at this developmental stage, before conducting future large-scale studies. There were few reports that examine the validity of measurement protocols themselves, and this point was one of a novelty of our study.

The mean fixation time percentage of all sequences was 81.0% and no significant difference among the four age groups. The criteria, “50% or more”, were met at all age groups, then the fixation time of all sequences in our study was enough to evaluate the gaze patterns data. In the following, we discuss the gaze patterns in each movie.

The movies I–V were human face stimuli. From birth, infants show interest in human faces^23^. Previous studies have suggested that children with ASD have less attention than children with TD to the human eyes^3,7,9,13,21^. However, a study that tracked ASD infants from 2 to 24 months, suggests that at 6 months fixations towards the eyes region are just beginning to decline, with maximal reductions in eye region fixation not occurring until 2 years^7^. Another study reported infants with ASD consistent gaze to the eyes region at 6 months, and greater amounts of fixation to the mouth during live interaction, predicted higher levels of expressive language at outcome and greater rates of growth^12^. The study using Gazefinder, also showed the moving mouth attracted the attention of young children with or without ASD^15^. Thus, infants in this age group, with or without ASD, possibly show more preference to human eyes than mouth, and are easy to pay attention by the moving mouth. Our results were consistent with previous reports and the participants identified human eyes and mouths on the monitor as targets of gaze starting at four months of age. The gaze behavior to the human eyes and mouth may not provide early biomarkers for autism around 1 year of age but can be used in predicting language acquisition.

The movie VI presented the biological motion. TD infants preferentially attend to biological motion within the first days of life, and preferential attention to biological motion is thought as a precursor to the capacity for attributing intentions to others^10,16,17,21^. A recent study that tested gaze patterns in repeatedly low ASD risk infants from 2 until 24 months of age, reported preference for upright biological motion that emerges by 3 months and continues to increase until 24 months^21^. In our study, infants from 4 to 11 months of age showed consistent preference to upright biological motion, but the aging effect, greater preference to biological motion by age, was not shown. This may depend on the quality of the stimulus movies and fewer sample sizes to detect the preference. Moreover, the previous study^21^ provided longer trial time (mean duration = 61 s), with analyses focused more on the second half of the trials, than our study (11 s), and it may not be sufficient time for interstimulus shifting and visual exploration for the infants. Previous studies reported, by 2–3 years old, children with ASD gaze at upright biological motion for shorter periods than did the TD children^10,16,17^, while other studie reported preschool children with ASD gazed at upright biological motion approximately the same as TD children^13^. The preference to biological motion under passive view conditions for children seems to be susceptible to the quality of stimuli and their age. Moreover, there have been few studies that reported changes in the gaze patterns of infants under 1 year old with ASD over time.

The movies VII and VIII simultaneously presented human and geometry. ASD children prefer to gaze at highly repetitive images (geometric patterns) rather than social images (human)^8,20^, and it was associated with increased symptom severity of ASD^8^. In previous research using Gazefinder to children between the ages of 1 year and 6 months to 2 years and 2 months, the M-CHAT and the fixation data of human and geometry (Movie VII and VIII), showed a significant correlation^14^. However, it has been known that TD individuals visually fixated on geometric images were enhanced with increasing age^8^ and the studies using Gazefinder reported the same trend^13,15,19^. One report showed the fixation to the human gradually decreased in the TD individuals and stabilized after around 5 years of age^15^. This trend is considered that during very early development TD infants and toddlers are strongly drawn to the human face^23^, while TD individuals may become able to alter their attention to non-social stimuli with increasing age^15^. Thus, enhanced gaze preference for geometric pattern is indicated as a developmental biomarker of ASD only in early childhood. In our study, in movie VII (same size), there was a positive association of age with gaze at human and a negative association with geometric patterns gaze, while it was not seen in movie VIII [small window] and participants showed consistent preference to gaze at human. Regarding movie VII, in 4–5 months group, the fixation percentage of geometric patterns were higher than human. This difference may be because the geometric patterns in Movie VIII occupies a relatively small area than Movie VII and then it was too small to identify the geometric patterns for early infants due to their limited visual acuity. Although, infants may show interest in geometric patterns at age 4–5 months, there were few studies that reported the preference of human and geometry. It was also needed to consider that the participants in our study did not accurately represent infants with TD.

Movie IX present joint attention that is a shared attention state between two individuals focused on an object or event of interest and is critically associated with language acquisition in TD children^22^ and with social deficits in ASD^11^. In our study, Group B (pointed object) indicated the emergence of joint attention, and the fixation percentage of pointed object increased with age. Moreover, the data of 20 participants who underwent two-times health checkups during the study period showed same trend. Infants develop joint attention around 10 months of age^11,12^, and we may measure the enhancement of joint attention skills with age. Joint attention has also been considered an important factor in learning and acquiring vocabulary in infants^22^, the stimuli may also be used as a quantitative predictor of vocabulary acquisition in infants.

## Limitations

This study was conducted in a small region, therefore, the generalizability of these results may be limited due to the potential impact of welfare and living conditions in this particular region. The number of subjects was small. We did not have prior assessments of high-risk infants or cohorts. In terms of epidemiology^2^, approximately 1% of participants were likely to receive a future diagnosis of ASD. Therefore, the data in this study did not accurately represent infants with TD.

The range of DQ was large (58–124%) and may be inaccurate due to missing values of some participants such as preterm infant. We divided participants into 5 groups according to the adjusted month of age with DQ (under 4 months, 4–5 months, 6–7 months, 8–9 months, 10 months or over) and conducted stratified analysis. There was no significant difference in the fixation time percentage of all sequences among the DQ groups (Kruskal–Wallis rank sum test p = 0.19). These results showed same trends to the examination at actual months of age. We need to perform the large study to clarify the relationship between the fixation time percentage and DQ.

## Conclusions

The fixation time percentage of all sequences was consistently high (mean 81%) from infants 4–5 months of age. In each stimulus movie, we confirmed the preference for human eyes and attention to mouth movements, preference for upright biological movements, and gaze at humans rather than geometric patterns. The enhancement of joint attention skills with age was also observed. Reproducibility with past findings was shown, and Gazefinder can be applied for infants from 4–5 months of age. Further studies using the same protocols are needed, while several details such as qualities of stimuli and duration time are also needed to be considered.

## Data Availability

The datasets generated and analysed during the current study are not publicly available because of our agreement with the caregivers of participants, but are available from the corresponding author on reasonable requests after gaining agreement from the caregivers of participants.

## Abbreviations

ASD: Autism spectrum disorder
TD: Typical development
DQ: Developmental quotient
